# Psychotic Experiences, Working Memory, and the Developing Brain: A Multimodal Neuroimaging Study

**DOI:** 10.1093/cercor/bhv181

**Published:** 2015-08-18

**Authors:** Leon Fonville, Kathrin Cohen Kadosh, Mark Drakesmith, Anirban Dutt, Stanley Zammit, Josephine Mollon, Abraham Reichenberg, Glyn Lewis, Derek K. Jones, Anthony S. David

**Affiliations:** 1 Section of Cognitive Neuropsychiatry, Department of Psychosis Studies, Institute of Psychiatry, Psychology and Neuroscience, King's College London, London, UK; 2 Department of Experimental Psychology, University of Oxford, Oxford, UK; 3 Cardiff University Brain Research Imaging Centre (CUBRIC), School of Psychology; 4 Institute of Psychological Medicine and Clinical Neuroscience, School of Medicine, Cardiff University, Cardiff, UK; 5 Centre for Academic Mental Health, School of Social and Community Medicine, University of Bristol, Bristol, UK; 6 Department of Psychiatry, Icahn School of Medicine, Mount Sinai Hospital, New York, NY, USA; 7 Division of Psychiatry, Faculty of Brain Sciences, University College London, London, UK

**Keywords:** ALSPAC, fMRI, neurodevelopment, psychotic experiences, working memory

## Abstract

Psychotic experiences (PEs) occur in the general population, especially in children and adolescents, and are associated with poor psychosocial outcomes, impaired cognition, and increased risk of transition to psychosis. It is unknown how the presence and persistence of PEs during early adulthood affects cognition and brain function. The current study assessed working memory as well as brain function and structure in 149 individuals, with and without PEs, drawn from a population cohort. Observer-rated PEs were classified as persistent or transient on the basis of longitudinal assessments. Working memory was assessed using the *n*-back task during fMRI. Dynamic causal modeling (DCM) was used to characterize frontoparietal network configuration and voxel-based morphometry was utilized to examine gray matter. Those with persistent, but not transient, PEs performed worse on the *n*-back task, compared with controls, yet showed no significant differences in regional brain activation or brain structure. DCM analyses revealed greater emphasis on frontal connectivity within a frontoparietal network in those with PEs compared with controls. We propose that these findings portray an altered configuration of working memory function in the brain, potentially indicative of an adaptive response to atypical development associated with the manifestation of PEs.

## Introduction

A number of psychiatric disorders first emerge during adolescence (Silva 1990; Paus et al. 2008) and are presumed to relate to the substantial social, cognitive, and physiological changes occurring during this period (Blakemore 2008). Psychiatric disorders with onset in childhood or adolescence further disrupt cognitive and social development, and there have been calls for new research into the underlying neurocognitive risk mechanisms during this period as well as the development of early and age-appropriate intervention approaches (Beddington et al. 2008).

Psychotic experiences (PEs), such as delusions, hallucinations, or thought interference, show that associations with a later psychiatric disorder (van Os et al. 2000) yet are also prevalent among the general population (van Os et al. 2009). The incidence of PEs is reportedly higher among children and adolescents than in adults (Cougnard et al. 2007; Kelleher et al. 2012), and persistence of PEs is a strong indicator of increased risk for later disorder, including psychosis (Kaymaz et al. 2012). Also, the mere presence of PEs has been linked with poor psychosocial outcomes, general psychopathology, self-harm, and cognitive impairment, even in the absence of a transition to psychosis (Nishida et al. 2010; Polanczyk et al. 2010; Barnett et al. 2012; Downs et al. 2013).

Impaired cognition is of particular interest, since lower childhood cognitive ability has been found to be predictive of PEs (Barnett et al. 2012; Niarchou et al. 2013). Furthermore, neurocognitive deficits present in adults, often in the domains of executive function, processing speed, and working memory (Simon et al. 2007; Fusar-Poli et al. 2012; Valli et al. 2012), increase in severity through prodromal phases toward clinical psychosis (Simon et al. 2007; Meier et al. 2014), and those who transition are found to have stronger neurocognitive deficits (De Herdt et al. 2013). Perhaps unsurprisingly, neuroimaging studies have extended the profile of frontal lobe dysfunction to prodromal populations both in terms of elicited activation (Fusar-Poli et al. 2007; Corlett and Fletcher 2012; Dutt et al. 2015) as well as the connectivity of underlying networks (Whalley et al. 2005; Allen et al. 2012; Jung et al. 2012; Diederen et al. 2013; Fryer et al. 2013; Schmidt et al. 2013; Orr et al. 2014; Schmidt, Smieskova, et al. 2014; van Lutterveld et al. 2014).

In the developing brain, prefrontal cortices are the last to reach structural maturity (Casey et al. 2005), a prolonged trajectory that is reflected in slowly developing executive functions and particularly working memory abilities (Casey et al. 2005; Blakemore 2008; Dumontheil and Klingberg 2012). Most studies of working memory have shown that during typical development from childhood to adulthood, memory capacity increases while brain activity becomes increasingly localized to a predominantly frontoparietal network (Casey et al. 2005; Klingberg 2006; Conklin et al. 2007). Advanced analysis techniques, such as dynamic causal modeling (DCM; Friston et al. 2003), have provided insights into the underlying dynamics of this network, showing that parietal regions are involved at an earlier stage of processing than frontal regions and increasing memory load modulates parietal-to-frontal connectivity (Ma et al. 2012; Dima et al. 2014). This network shows aberrant connectivity in individuals with psychosis and, to a lesser extent, those at high risk for psychosis [see Schmidt, Diwadkar, et al. (2014) for review]. Interestingly, this same pattern has been reported with regard to frontotemporal connectivity (Lawrie et al. 2008; Crossley et al. 2009; Allen et al. 2010).

Though limited, the current literature on PEs has also pointed to changes predominantly in frontal and temporal regions in terms of fMRI blood oxygenation level-dependent (BOLD) response (Jacobson et al. 2010; Corlett and Fletcher 2012), functional connectivity (Diederen et al. 2013; Orr et al. 2014; van Lutterveld et al. 2014), gray matter volume (Jacobson et al. 2010; Modinos et al. 2010; Cullen et al. 2013), and white matter microstructure (Jacobson et al. 2010). However, to date, only one small study has employed a simple multimodal approach (Jacobson et al. 2010) and more studies are needed to capture both the structure of the brain as well as the underlying neural dynamics.

The aim of this study was to shed light on the neuropsychological profile of those with PEs by administering a working memory task in combination with functional and structural brain imaging. We hypothesized that PEs during late adolescence affect neurodevelopmental trajectories, and we would be able to measure an impact on brain function in those with PEs in terms of 1) reductions in BOLD response within a working memory network and 2) differences in frontoparietal network configuration, or modulatory connections, similar to those found in individuals at high risk for psychosis (Schmidt et al. 2013; Schmidt, Smieskova, et al. 2014). Furthermore, persistence of PEs from late adolescence into adulthood would have a more profound impact, and we hypothesized that this would be demonstrable in terms of working memory performance and BOLD signal reductions. Finally, we undertook to examine regional gray matter volume as a potentially confounding factor in relation to brain function.

## Methods

### Participants

All participants were part of the Avon Longitudinal Study of Parents and Children (ALSPAC; http://www.bristol.ac.uk/alspac/). A total of 4724 young adults, out of an initial cohort of around 14 000 births, were assessed with the Psychosis-Like Symptom interview (PLIKSi) at age 18 and 433 (9.2%) individuals were rated as having suspected or definite PEs (Zammit et al. 2013). From this sample, 165 participants (93 controls and 72 with suspected or definite PEs) were reassessed using the PLIKSi and underwent functional and structural MRI at age 20. Participants' informed consent was obtained before imaging and ethical approval was given by the Cardiff University School of Psychology Ethics Committee and the ALSPAC Ethics and Law Committee.

**Figure 1.**
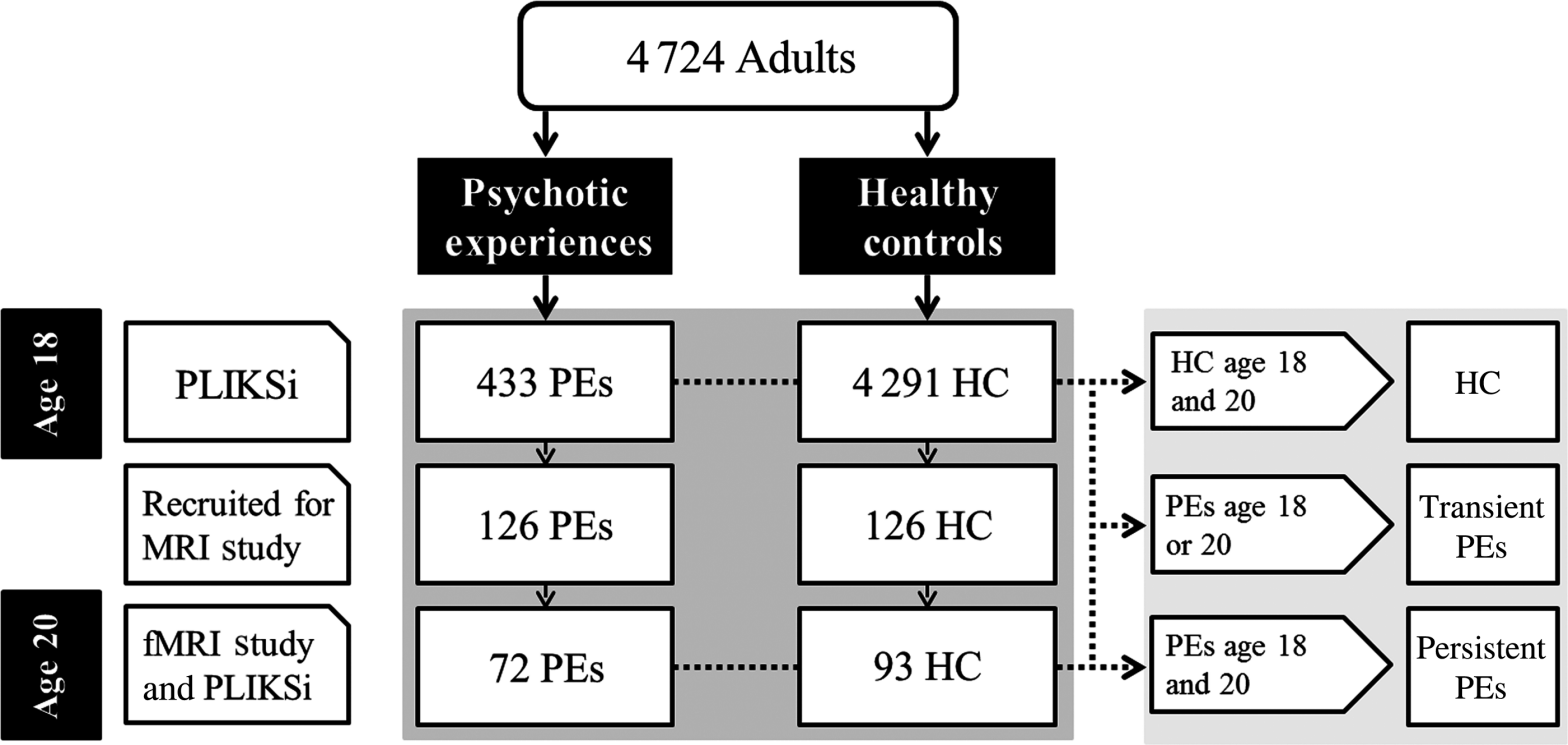
Flowchart depicting ALSPAC participants with regard to PLIKSi at ages 18 and 20.

A total of 16 participants were excluded from analyses due to missing PLIKSi data at both time-points, task performance at or below chance level, or technical issues with fMRI data preprocessing. Participants were divided into 3 groups based on PLIKSi rating at both time-points (Fig. 1). Those who were rated as having PEs at both age 18 and age 20 were considered persistent PEs, those with PEs at either age 18 or 20 were considered transient PEs, and those rated as not having PEs at either time-points as healthy controls (HCs). In total, there were 35 participants with persistent PEs, 36 with transient PEs, and 78 HCs. Out of the 36 with transient PEs, only 9 participants (25%) had been rated as having PEs at age 20 and the other 27 participants (75%) were only rated as having PEs at age 18.

Missing values were estimated using a regression imputation method (Buck 1960) across the entire ALSPAC cohort (*n* = 13 971). Groups differed in childhood IQ (taken at age 8 using the Wechsler Intelligence Scale for Children), which was driven by differences between persistent PEs and HC, but were similarly distributed in terms of reported dominant hand (Table 1).

**Table 1:** Description of sample: Demographics, childhood IQ, and handedness

	Controls	Transient PEs	Persistent PEs	Test statistic
*n*	78	36	35	
Age	20 (1)	20 (0)	20 (1)	
Gender				
Male	31 (40%)	7 (19%)	8 (23%)	*χ*^2^ = 6.092, *P* = 0.048
Female	47 (60%)	29 (81%)	27 (77%)	
Handedness				
Right	61 (78%)	27 (75%)	29 (83%)	
Left	14 (18%)	7 (19%)	5 (14%)	
No dominant hand	3 (4%)	2 (6%)	1 (3%)	
IQ at age 8	114.5 (22)	107.5 (15)	105.0 (15)	*χ*^2^ = 10.769, *P* = 0.005

Note: Age and IQ are given in median (interquartile range), and gender and handedness are given as frequency (percentage).

### Psychotic Experiences

PEs were assessed using the PLIKSi, a semi-structured interview covering the occurrence of visual hallucinations, auditory hallucinations, delusions (being spied on, persecution, thoughts being read, reference, control, grandiose ability, and other unspecified delusions), and thought interference (thought broadcasting, insertion, and withdrawal) in the past 6 months, and were administered at ages 18 and 20. Respondents were asked 12 core questions, 7 derived from the Diagnostic Interview Schedule for Children, Version IV (DISC-IV) and 5 from the Schedules for Clinical Assessment in Neuropsychiatry (SCAN), version 2, and clinical cross-questioning and probing was used to establish the presence or absence of any experiences. Interviewers rated experiences as definitely present, suspected to be present or absent, and unless a credible example was given, responses were rated down to suspected instead of definitely present. An overview of the reported PEs in each group at ages 18 and 20 is given in Supplementary Table 1.

### *N*-Back Task

A letter variant of the *n*-back task was used. Participants were instructed to press a button with their index finger when the letter that was presented on the screen was identical to the one they saw *n* trials earlier, where *n* can be 1, 2, or 3. During 0-back testing, participants were instructed to press the button whenever the letter X was presented on the screen. Each condition was presented 3 times in a pseudorandom order in blocks of 14 items; each item lasted 2 s, and was preceded by a 3-s written instruction on the screen. During each block, there were 3 correct combinations, giving a maximum of 9 correct responses per condition. Including the instruction, each block was 31 s long making the total duration of the *n*-back task 372 s. Task performance was measured in terms of reaction time and by the sensitivity index *d*′, computed as *d*′ = *Z*_HIT_− *Z*_FA_, where FA reflects false alarms. Hit and false alarm rates of 0 or 1 were adjusted as described in Haatveit et al. (2010). The highest possible *d*′ score was 3.85 and the lowest was −3.85. Owing to non-normality of the data, the Kruskal–Wallis test was used to test for group differences and pairwise post hoc comparisons were performed using Dunn–Bonferroni correction. To test for any group × gender interaction effects, an adjusted rank transformation (ART) was applied as described in Leys and Schumann (2010). In short, this approach subtracts the marginal means from each individual's score and assigns a rank to the adjusted score. A factorial ANOVA is then performed on the ranked data to test for interaction effects.

### MRI Acquisition

Imaging data were acquired at the Cardiff University Brain Imaging Centre (CUBRIC) on a 3-T General Electric SIGNA HDx (GE Medical Systems, Milwaukee, WI, USA) using an 8-channel head coil for radiofrequency reception.

Changes in BOLD were measured using *T*_2_*-weighted gradient-echo echo-planar images along the axial plane parallel to the anterior commissure–posterior commissure (AC–PC) line [repetition time (TR) = 2000 ms, echo time (TE) = 30 ms, flip angle = 75°, field of view = 240 × 240 mm, resolution = 3.75 × 3.75 × 3.5 mm].

A high-resolution, fast-spoiled gradient-echo *T*_1_-weighted isotropic image was acquired with slices parallel to the AC–PC line (TR = 7808 ms, TE = 2988 ms, inversion time = 450 ms, flip angle = 20°, field of view = 256 × 256 mm, resolution = 1 mm^3^) to improve functional image registration to the standard space and for investigation of gray matter.

### Preprocessing

Statistical parametric mapping (SPM) was performed using SPM8 (www.fil.ion.ucl.ac.uk/spm). Functional imaging data were realigned and resliced using the first image as a reference. Slice timing correction was applied and each individual's fMRI time-series was coregistered to a *T*_1_-weighted structural image using the mean image as the reference and normalized mutual information as the cost function. All *T*_1_-weighted images were segmented using default tissue probability maps of gray and white matter before importing the segmentation parameters in DARTEL (Ashburner 2007) and producing rigidly aligned gray matter images. A study-specific mean image template was reiteratively created, and the final template was affine transformed to the template defined by the Montreal Neurological Institute (MNI). Resulting deformations were applied to transform the segmented gray matter images and fMRI time-series to the MNI template, and an 8-mm full-width half-maximum Gaussian smoothing kernel was applied.

### fMRI Analysis

At the first level, with the exception of 0-back which was used as an implicit baseline, the onsets of each condition were convolved with a canonical hemodynamic response function and serial correlations were modeled as an autoregressive process. Six movement parameters were added as nuisance covariates. A main effect for each explicit condition (1-back, 2-back, and 3-back) was modeled and entered into a random-effects analysis at the second level. A main effect of task was computed by collapsing groups and conditions, and the increase in BOLD response was used as an activation mask at the second level. Linear and quadratic trends in BOLD response with increasing task complexity were tested across groups. The 3 groups were then compared on each condition using one-way ANCOVA's, controlling for gender, premorbid IQ, and their performance (*d*′). Finally, a group × task interaction analysis was performed. Statistical significance was inferred at a threshold of *P* < 0.05 after family-wise error (FWE) correction.

### Voxel-Based Morphometry (VBM) Analysis

Voxel-wise comparison of modulated *T*_1_-segmented gray matter images of the 3 groups was performed using a one-way ANCOVA, controlling for gender, and premorbid IQ. As with fMRI, a threshold of *P* < 0.05 FWE-corrected was used to assess statistical significance.

### DCM Analysis

Underlying dynamics of frontoparietal connectivity were analyzed using DCM version 10 (Friston et al. 2003). First, regional time-series derived from first-level general linear modeling were extracted from spherical volumes of interest (VOI), 6 mm in diameter, from the nearest subject-specific local maxima near the peak of activation overlap in the bilateral middle frontal gyrus and posterior parietal lobules (as depicted in Supplementary Fig. 2) using the first eigenvariate of voxels above a subject-specific threshold of *P* < 0.05 uncorrected. Second, all models were specified using the same intrinsic connections, allowing reciprocal frontoparietal connections within each hemisphere and interhemispheric connections between frontal and parietal regions. Visual input was specified as reaching the parietal lobules bilaterally first after initial cortical reception. Specification of model configuration, matched with previous literature (Schmidt et al. 2013; Schmidt, Smieskova, et al. 2014), was based on 3 possible directions of modulation from the input and could be forward parietal-to-frontal, backward frontal-to-parietal, or both. In each of these directions, there were 4 possible configurations by taking interhemispheric modulation into account: A lack of interhemispheric modulation, only frontal interhemispheric modulation, only parietal interhemispheric modulation, or both frontal and parietal interhemispheric modulation. In total, this leads to 12 different models that test different modulatory effects and cover all physiologically possible connections. These models were fit to the 2-back and the 3-back, respectively, compared with the 0-back baseline. Bayesian model selection (BMS) was used to compute both the exceedance and expected posterior probabilities at the group level (Stephan et al. 2009). The exceedance probability, that is the probability that a certain model is more likely than the others, was used to infer the best model fit in each group.

## Results

### *N*-Back Performance

Groups differed significantly in performance, as measured using the sensitivity index *d*′, on the 1-back and the 2-back. Performance was poorer in persistent PE compared with HC, with transient PE not being significantly different from either group. Using the ART, a group × gender interaction effect was found on the 0-back and the 3-back. Splitting the dataset by gender revealed a difference between males with persistent PEs compared with male controls on the 0-back, but no other differences were found. Average performance on each condition per gender in each group is presented in Supplementary Figure 1. Those with persistent PE had a faster reaction time on the 3-back compared with HC with neither group differing from transient PE. No other group differences were present and there was no indication of a group × gender interaction effect on reaction time. Performance measures are summarized in Table 2.

**Table 2:** Performance summary for each group given as median (interquartile range)

	Controls	Transient PEs	Persistent PEs	Test statistic	Group × gender interaction
*d*′ _(Range −3.85 to 3.85)_					
0-Back	3.85 (0.00)	3.85 (0.38)	3.85 (0.37)	*χ*^2^(2, 149) = 7.967, *P* = 0.019	*F*_2,143_ = 23.074, *P* < 0.001
1-Back	3.85 (0.00)	3.85 (0.37)	3.48 (0.37)^a^	*χ*^2^(2, 149) = 10.779, *P* = 0.005	
2-Back	3.48 (0.77)	3.48 (1.16)	3.10 (1.54)^a^	*χ*^2^(2, 149) = 8.139, *P* = 0.017	
3-Back	2.64 (1.02)	2.60 (0.62)	2.31 (1.00)		*F*_2,143_ = 3.912, *P* < 0.022
Reaction time (ms)					
0-Back	470.45 (90.61)	471.83 (103.08)	424.33 (133.89)		
1-Back	532.89 (133.87)	530.67 (150.62)	512.44 (144.34)		
2-Back	622.26 (173.39)	606.50 (201.62)	575.25 (187.58)		
3-Back	722.86 (232.85)	655.18 (284.53)	655.67 (175.12)^a^	*χ*^2^ (2, 149) = 6.662, *P* = 0.036	

^a^Post hoc test revealed a significant decrease in persistent PE versus controls.

### fMRI Analysis

#### Working Memory Network

**Figure 2.**
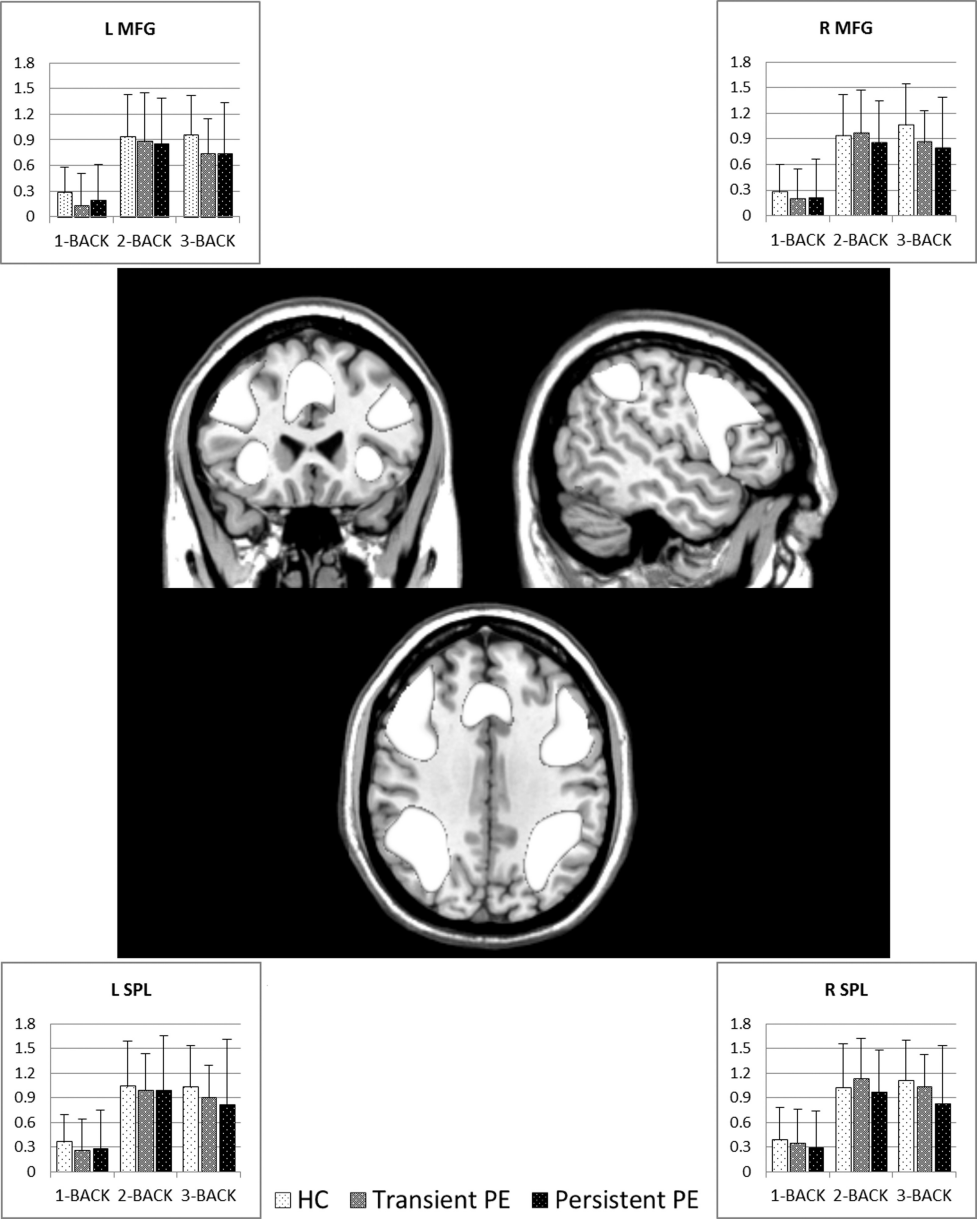
Center: Task-related increases in BOLD response upon collapsing both groups and conditions used as a functionally defined working memory network. The average BOLD response to each condition has been extracted and plotted for the left middle frontal gyrus (top left), right middle frontal gyrus (top right), left superior parietal lobule (bottom left), and right superior parietal lobule (bottom right).

The main effect of each condition (1-back, 2-back, and 3-back) and group membership were collapsed to compute a main effect of task. This contrast revealed a consistent pattern of bilateral activation predominantly in the middle frontal gyrus and superior parietal lobule, as well as in the insula and supplementary motor area extending into the anterior cingulate cortex (Fig. 2). An overview of regions showing task-elicited increases in activation is given in Supplementary Table 2.

The overall activation was used as a mask of a working memory network for further analyses. A one-way whole-brain ANOVA was performed to test if the groups recruited brain regions differently on the task. No differences in BOLD response were found between groups when collapsing conditions.

Further analyses comparing groups on each condition (1-back, 2-back, and 3-back, respectively) were performed utilizing the working memory mask. No group differences in BOLD response were found on any condition.

Linear and quadratic trend analyses were performed on the whole sample to assess the effect of cognitive load and revealed almost identical patterns. There was a strong increase in BOLD response from 1-back to 2-back followed by a slight increase or decrease depending on the contrast weights (summarized in Supplementary Tables 3 and 4), as is illustrated in Figure 2 for the bilateral middle frontal gyrus and superior parietal lobule.

A group × task interaction analysis did not reveal any significant interactions at *P* < 0.05 FWE-corrected.

### VBM Analysis

Voxel-wise, whole-brain analysis of local gray matter volume revealed no differences between the groups or any correlation between local gray matter volume and premorbid IQ across all participants. Limiting voxel-wise comparisons to the functionally defined working memory network (as depicted in Fig. 2) did not reveal any differences in gray matter volume between the 3 groups either.

#### Volumes of Interest

Regional time-series were extracted from spherical volumes centered on subject-specific local maxima in the frontal and parietal regions of the brain as VOI. Group local maxima coordinates across the task were entered as initial reference points for each VOI and are described in Table 3. Deviations from these reference coordinates to subject-specific local maxima were limited to the overlap in activation between the 3 groups within the working memory network mask (see Supplementary Fig. 2).

**Table 3:** Cluster properties of VOI utilized in DCM comprised of brain regions that show an increase in BOLD signal during the task compared with the baseline and overlap across participants in all 3 groups in BOLD signal increases during the task

Region	Size (voxels)	MNI coordinates			*t*-value	*P*-value (FWE-corrected)
		*X*	*Y*	*Z*		
R superior parietal lobule	15 468	46	−46	47	22.46	<0.001
L superior parietal lobule	12 012	36	−47	38	19.70	<0.001
R middle frontal gyrus	13 700	30	6	54	18.31	<0.001
L middle frontal gyrus	9440	−27	1	54	17.64	<0.001

MNI, Montreal Neurological Institute; FWE, family-wise error correction.

### DCM Analysis

Model comparison was done using random-effects BMS and was performed across all groups and within each group.

#### Model Fit for the 2-Back

**Figure 3.**
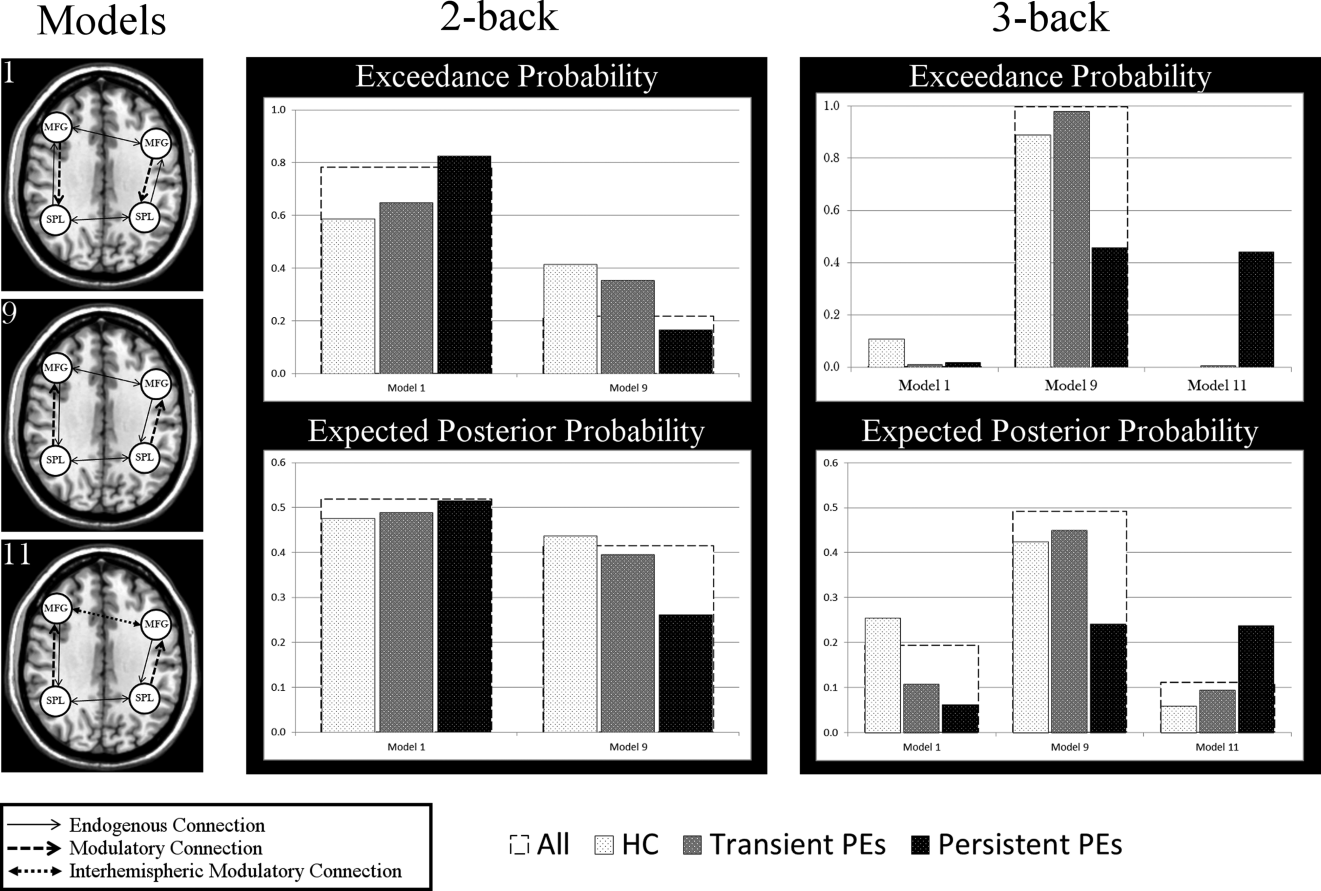
Illustrations of winning models (left) and the probabilities associated with these models (range 0–1) given for the total sample and for each group on both the 2-back and 3-back conditions.

Model 1 (frontal-to-parietal modulation without any interhemispheric modulation, see the top left of Fig. 3) was the best-fitting model with an exceedance probability (i.e., likelihood of that model best explaining the observed data) of 78% followed by model 9 (parietal-to-frontal modulation without any interhemispheric modulation, see the bottom left of Fig. 3) with a probability of 22%. When looking within each group, the same frontal-to-parietal modulation model was the best-fit but the exceedance probability decreased from persistent PEs (82%) to transient PEs (65%) to HCs (59%), while that of parietal-to-frontal modulation increased from persistent PEs (18%) to transient PEs (35%) to HCs (41%; Fig. 3).

#### Model Fit for the 3-Back

Model 9 (parietal-to-frontal modulation without interhemispheric modulation) was the best-fit to the data with an exceedance probability of 99.8%. This finding was repeated within groups for the HC (at 89%) and for transient PEs (at 98%). Though this was still the winning model in persisting PEs at 46%, the second best model 11 (parietal-to-frontal modulation with frontal interhemispheric modulation) had a similar exceedance probability of 44%.

## Discussion

The aim of this study was to assess working memory function in individuals with PEs using functional and structural MR imaging, as well as a letter variant of the *n*-back task. We hypothesized that PEs during late adolescence represent a deviation from typical development and would affect working memory function. Additionally, abnormal persistence of PEs would lead to greater alterations in development and this would be demonstrable as a dose–response relationship between PE duration and both poorer task performance and reduced BOLD signal. There was indication of differences in performance and further analysis revealed that those with persistent PEs performed worse than HC, while there was no evidence that those with transient PEs differed from either group. However, there were no differences between groups in recruitment of brain regions or BOLD signal intensity in a functionally defined working memory network. Additionally, analyses of gray matter volume in the brain revealed no differences in brain structure between the groups. Further testing of effective connectivity did not support our hypotheses of qualitative differences in frontoparietal network configuration between groups. The same model of frontal-to-parietal modulation was the best-fit to the data on the 2-back in all groups, but crucially, in the light of the lack of other differences between the groups, the probability dropped in a dose–response fashion from persistent PEs to transient PEs, to HCs. The opposite pattern was found in terms of parietal-to-frontal modulation, which was highest in HCs, lowest in persistent PEs, and intermediate in the transient PE groups. On the 3-back, we found a strong consistent pattern of parietal-to-frontal modulation in all groups, but in persistent PEs there was an additional presence of frontal interhemispheric modulation. Below we discuss the significance of these findings.

### Poorer Working Memory Performance in Persistent PEs

Reduced cognitive ability has been reported in PEs (Barnett et al. 2012; Niarchou et al. 2013), and poorer performance in domains, such as working memory, attention, and processing speed, at ages 8–11 has been associated with PE in the ALSPAC cohort at age 12 (Niarchou et al. 2013). However, it is unclear whether reduced cognitive ability is due to a developmental lag, or to a deviation from typical development due to PEs. The current sample of young adults at age 20 does demonstrate some reduction in working memory function, but only those with persisting PEs were statistically discernible from HCs. Previous studies have not made distinctions regarding the duration of PEs. Additionally, there was some evidence of a group × gender interaction effect on working memory performance. However, these findings should be interpreted with caution due to the small number of males present in both groups with PEs. Gender differences have been described (Maric et al. 2003; Johns et al. 2004; van Os et al. 2009; Dominguez et al. 2010), but findings differ and further research on the potential effects of gender on PEs is required. It should be noted that studies using the *n*-back in combination with fMRI in prodromal states often do not find differences in raw performance between those deemed “at-risk” and controls (Seidman et al. 2006; Crossley et al. 2009; Fusar-Poli et al. 2011; Smieskova et al. 2012). The fact that the performance of those with transient PEs lies intermediate to persistent PEs and HCs lends further credence to the hypothesis that abnormal persistence of PEs is associated with a more profound and potentially pathological effect on working memory function.

### Similar Brain Function in Response to Working Memory Demands

There was no indication of differences in the recruitment of brain regions involved with working memory, and the groups did not differ in BOLD response on the *n*-back task. Previous fMRI studies reporting differences in cognitive function in those with PEs either studied children aged 9–11, without further controlling for other confounders affecting development (Jacobson et al. 2010), or only found associations between BOLD response and magical ideation (Corlett and Fletcher 2012). Other studies have highlighted aberrations in functional connectivity using resting-state fMRI, implicating reductions in frontotemporal connectivity (Diederen et al. 2013) and changes in connectivity between the default mode network and temporal regions (Orr et al. 2014; van Lutterveld et al. 2014). As before, it should be noted that only one of the aforementioned studies looked at PEs in general (Orr et al. 2014), while the others assessed functional connectivity solely only in those with auditory verbal hallucinations (Diederen et al. 2013; van Lutterveld et al. 2014) and all of these studies used very small samples. The current study focused on working memory and the frontoparietal network, but it is possible that other networks in the brain do show differences. A recent study of structural network topology in the ALSPAC neuroimaging cohort revealed changes in graph theory metrics, including a reduction in global efficiency and density, as well as reductions in local efficiency in cingulate, parietal, occipital, and frontal regions (Drakesmith et al. 2015). However, no distinction was made between durations of PEs in that study.

### Gray Matter Volume in PEs

Contrary to previous studies of brain volume in PEs, which found increased temporal gray matter volume (Jacobson et al. 2010; Modinos et al. 2010; Cullen et al. 2013), there were no differences in underlying gray matter volume in a whole-brain analysis between the groups and gray matter volume was not added as a potential confounder to our analyses. However, both Jacobson et al. (2010) and Cullen et al. (2013) examined gray matter volume in developing children, and Modinos et al. (2010) reported a positive correlation between gray matter volume and higher levels of PEs assessed using self-report measures. Negative correlations between frontal and temporal cortical thickness and IQ have been found in early childhood, followed by a slower decline in cortical thinning in late childhood and adolescence (Shaw et al. 2006). The current sample with PEs showed lower childhood IQ, but gray matter volume in young adulthood showed no relation to childhood IQ. Nevertheless, we cannot rule out variations in cortical maturation during development.

### Frontoparietal Network Configurations

This is the first study to investigate effective connectivity in non-clinically identified, non-help-seeking young people with PEs, and the same frontoparietal network underlying working memory function was identified in all the 3 groups. The variance in BOLD signal in this network during the 2-back condition was best-explained as a feedback model where the increase in BOLD signal in the parietal lobules, which is propagated to the frontal lobes, is modulated by a backward connection from the frontal lobes to the parietal lobules. Nevertheless, the probability of this frontal-to-parietal modulation model being the best-fit to the data decreased in a linear fashion from persistent PEs to HCs, with transient PEs in between. In HCs, the variance in BOLD signal was captured almost equally well as a signal increase in the parietal lobules that is propagated to the frontal lobes and modulates the increase in BOLD signal. The probability of this parietal-to-frontal modulation model being the best-fit to the data decreased from HCs to transient PEs to persistent PEs. These findings suggest a greater role for the frontal lobes in those with PEs, particularly in persistent PEs. During the 3-back, there was a clear shift across all groups toward parietal-to-frontal configuration but, unlike HC and transient PE groups, in persistent PEs the BOLD signal within this network was captured almost equally well with or without the presence of interhemispheric frontal modulation.

These differences in network configurations, in light of similar recruitment of brain regions and no differences in either BOLD signal strength or gray matter volume, reflect differences in the underlying temporal dynamics of distinct regions and highlight the importance of studying cognitive functions in terms of connectivity. Throughout adolescence and into adulthood, there are ongoing changes in working memory circuitry which reflect neural maturation and further specialization toward task-specific processing (Luna et al. 2010). These changes are associated with improvements in cognitive function and reflected in behavioral performance. Considering the minor differences in performance on the 2-back, these differences in working memory dynamics could be interpreted as a subtle delay in maturation of a fully formed network whereby those with PEs show a greater dependence on top-down neural signaling from frontal areas. The 3-back condition represents a greater demand in cognitive resources and a parietal-to-frontal configuration was found to clearly best-fit the data. However, in persistent PEs, there was an additional role for the frontal lobes in terms of interhemispheric communication. In addition to strong convergence of network model configuration, there were no differences in performance on the 3-back. A previous work by Dima et al. (2014) on the *n*-back task in healthy volunteers has similarly found a shift toward parietal-to-frontal configurations with an increase in cognitive demands from the 1-back up to the 3-back, and Ma et al. (2012) also reported an enhanced connection from parietal to frontal regions at higher digit loads. Taken together, it seems that at increasing cognitive demands, a parietal-to-frontal configuration is being utilized, but this is less strongly the case in those with PEs, in particular persisting PEs.

Refinements in macro- and microstructure of this network occur throughout adolescence before stabilizing during adulthood (Shaw et al. 2008; Ostby et al. 2011) and lead to a decreased reliance on frontal areas as more specialized posterior regions become increasingly recruited (Luna et al. 2010). In our study, subtle changes can be observed at lower cognitive load in working memory circuitry and coincide with poorer performance but are less pronounced when all groups are struggling. The question arises if these differences are indicative of a developmental lag, a greater reliance on frontal top-down signaling in those with PEs that can be observed when their peers are able to perform the task adequately, or indicative of atypical wiring within this network that leads to a more prominent role for the frontal lobes. The few fMRI studies on PEs have reported greater connectivity in those with PEs than controls (Diederen et al. 2013; Orr et al. 2014), in contrast to the majority of studies in high-risk and psychosis which report a decrease in connectivity regarding the frontal lobes (Pettersson-Yeo et al. 2011). Specifically, Orr et al. (2014) report hyperconnectivity between frontal and parietal areas of the brain in PEs compared with controls. In this sense, the prominent role of the frontal lobes could be considered a protective factor or resilience in response to a deviation in typical development that is associated with the manifestation of PEs (Orr et al. 2014; Johnson et al. 2015). However, due to the cross-sectional nature of the imaging data, we are unable to comment on changes in these patterns over time.

### PEs and Neurodevelopmental Risk Factors

While the current findings illustrate differences in working memory function and brain network configuration in those with persistent PEs compared with HCs, it is uncertain how this relates to the very phenomena that characterize these groups. More specifically, does the presence of PEs cause alterations in working memory function or do changes in cognitive development lead to the manifestation of PEs? Though trajectories vary for specific cognitive functions, numerous studies on premorbid neuropsychological functioning have reported IQ deficits prior to the onset of schizophrenia (Woodberry et al. 2008; Reichenberg et al. 2010; Dickson et al. 2012; Meier et al. 2014), which do not seem to progress with age nor in the presence of prodromal psychotic symptoms (Woodberry et al. 2008). Neuroimaging studies also report a progressive decline in gray and white matter structures after the onset of psychosis compared with the prodromal stage (Pantelis et al. 2005; Peters and Karlsgodt 2015). As such, it seems unlikely that the emergence of PEs is the driving force behind differences in working memory function. Instead, early neurodevelopmental risk factors, such as lower childhood IQ, may play a role in the manifestation of PEs and, in turn, psychiatric disorders.

### Strengths and Limitations

This study utilized a multimodal approach to assess working memory function in PEs by examining the overall structure of gray matter and the regional blood flow of brain regions recruited in working memory. Additionally, DCM allowed for analysis of connectivity within this identified network to elucidate more subtle differences between groups. However, this is limited to the subject-specific regional activation and is not a measure of structural connectivity in the brain. The administration of a stringent semi-structured interview rated by trained observers, rather than a self-report measure to define PEs is one of the main strengths of this study. Overestimation of psychotic phenomena due to self-report measures limits their usefulness in aiding our etiological understanding of psychotic disorders. In the current sample, we assessed the presence of PEs over the past 6 months at ages 18 and 20 and as such we cannot say with absolute certainty that persistent PEs are stable from age 18 to 20. A close look at the rated PEs in this group did suggest, however, that the same type of symptoms is being reported at each time point, but more research on persistence of PEs is required. Similarly, the definition of transient PEs in the current study did not differentiate between those rated as having PEs at age 18 or 20. Currently, it is uncertain whether there is a difference between those who have recently had PEs and those who now have PEs.

The use of a well characterized, epidemiologically ascertained sample with detailed demographic and psychosocial assessments allowed for well-matched samples in terms of age, gender, and handedness. While the current sample includes more females than males, Zammit et al. (2013) reported that, in the ALSPAC cohort, females were more likely than males to have PEs. Similarly, other population-based studies have reported gender differences in psychotic phenomena, but overall findings have been inconsistent (Maric et al. 2003; Johns et al. 2004; van Os et al. 2009; Dominguez et al. 2010).

### Future Directions

The availability of longitudinal ratings of PEs over 2 years in a homogenous cohort allowed for a thorough assessment of brain function in PEs and while no significant differences were found in the localization and overall strength of activation, there was a meaningful deviation from HCs in terms of connectivity and performance in those with persisting PEs. It is unknown what causes persistence of PEs in some, while in others these symptoms dissipate over time. Our findings further highlight the impact of persistence and the intermediate status of those with transient PEs. What differentiates those who continue and those who cease to have PEs is of utmost importance for improving our understanding of both the phenomenology of PEs and the psychosis continuum. Having said that, as a group those with persistent PEs are at increased risk of developing a psychiatric disorder, some indeed the majority are likely to remain illness-free with other bio-psychosocial factors determining resilience, functional decline, or even transition to psychosis (Bak et al. 2005; Hanssen et al. 2005; Krabbendam, Myin-Germeys, Bak, et al. 2005; Krabbendam, Myin-Germeys, Hanssen, et al. 2005; Zammit et al. 2013). This further highlights the need for longitudinal research in PEs in population-based samples, such as ALSPAC, to understand the role that mediating factors play and to identify factors that are predictive of transition to psychiatric disorders. Finally, although the mere presence of PEs can be considered a deviation from typical development, a closer look at the type of PE as well as frequency and severity could clarify some of the heterogeneity of outcomes within those with PEs.

## Supplementary Material

Supplementary material can be found at: http://www.cercor.oxfordjournals.org/.

## Funding

The work was funded by a grant from the UK Medical Research Council. ASD and AR are also supported by the National Institute of Health Research (NIHR) Biomedical Research Centre at the South London and Maudsley NHS Foundation Trust and Institute of Psychiatry, Psychology and Neurosciences, King's College London. Funding to pay the Open Access publication charges for this article was provided by King's College London.

## Supplementary Material

Supplementary Data
