# Supplementary material for: Psychotic Experiences, Working Memory, and the Developing Brain: A Multimodal Neuroimaging Study
Source: Cereb Cortex. 2015 Aug 18;25(12):4828–38. doi: 10.1093/cercor/bhv181 (PMC4635922; doi:10.1093/cercor/bhv181)
Supplement: Supplementary Data [file supp_25_12_4828__index.html]

Psychotic Experiences, Working Memory, and the Developing Brain: A Multimodal Neuroimaging Study — Psychotic Experiences, Working Memory, and the Developing Brain: A Multimodal Neuroimaging Study — Supplementary Data 

# Psychotic Experiences, Working Memory, and the Developing Brain: A Multimodal Neuroimaging Study

## Supplementary Data

Supplementary Data

- Supplementary Figure 1 - tif file
- Supplementary Figure 2 - tif file
- Supplementary Tables - doc file
