# Supplementary figures and images for: Psychotic Experiences, Working Memory, and the Developing Brain: A Multimodal Neuroimaging Study
Source: Cereb Cortex. 2015 Aug 18;25(12):4828–38. doi: 10.1093/cercor/bhv181 (PMC4635922; doi:10.1093/cercor/bhv181)

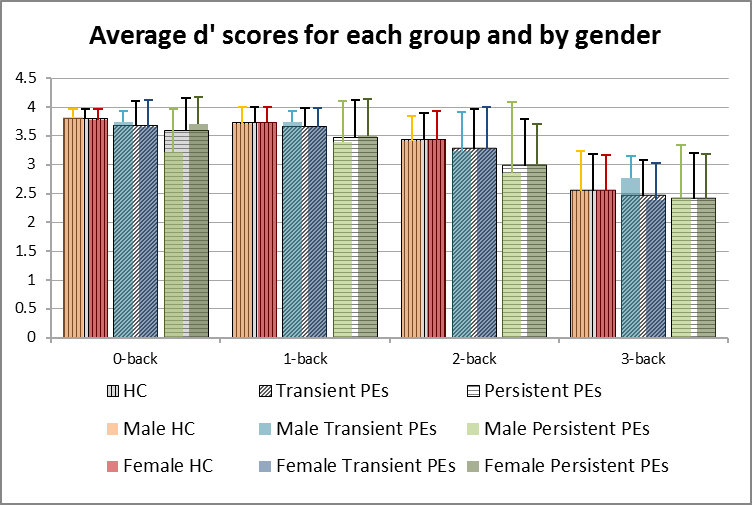

Supplement: Supplementary Data [file supp_bhv181_bhv181supp_fig1.tif]

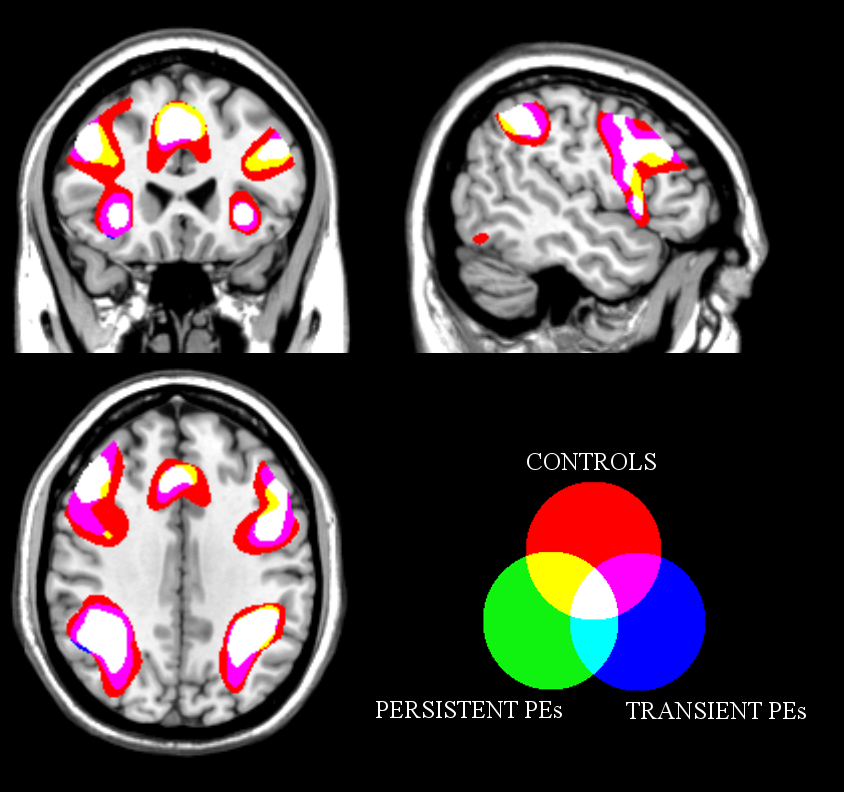

Supplement: Supplementary Data [file supp_bhv181_bhv181supp_fig2.tif]
