# Supplementary material for: Psychotic Experiences, Working Memory, and the Developing Brain: A Multimodal Neuroimaging Study
Source: Cereb Cortex. 2015 Aug 18;25(12):4828–38. doi: 10.1093/cercor/bhv181 (PMC4635922; doi:10.1093/cercor/bhv181)
Supplement: Supplementary Data [file supp_bhv181_bhv181supp_tables.doc]

Supplementary Table 1. Reported psychotic experiences (PEs) at ages 18 and 20 in each group. Individuals with transient PEs had PEs at either age 18 or age 20. Those with persistent PEs had PEs at both age 18 and age 20.

|  | **Transient PEs (n = 36)** | | **Persistent PEs (n = 35)** | |
| --- | --- | --- | --- | --- |
|  |  |  |  |  |
| Age | 18 | 20 | 18 | 20 |
| n | 27 (75%) | 9 (25%) | 35 (100%) | 35 (100%) |
| Rated Psychotic Experiences | | | | |
| Thought Interference (TI) | 3 (11.1%) | 0 (0%) | 1 (2.9%) | 1 (2.9%) |
| Delusions (DL) | 2 (7.4%) | 7 (77.8%) | 4 (11.4%) | 2 (5.7%) |
| Hallucinations (HAL) | 14 (51.9%) | 1 (11.1%) | 20 (57.1%) | 20 (57.1%) |
| TI + DL | 0 (0%) | 0 (0%) | 0 (0%) | 0 (0%) |
| TI + HAL | 5 (18.6%) | 1 (11.1%) | 5 (14.3) | 5 (14.3) |
| DL + HAL | 2 (7.4%) | 0 (0%) | 2 (5.7%) | 3 (8.6%) |
| TI + DL + HAL | 1 (3.7%) | 0 (0%) | 3 (8.6%) | 4 (11.4%) |

Supplementary Table 2. Task-elicited increases in BOLD response across conditions and groups. Statistics are provided for cluster peak coordinates and local maxima in each cluster that are more than 8mm apart are noted underneath.

| **Region** | **Size (voxels)** | **MNI** |  |  | **t-value** | **p-value  (FWE corrected)** |
| --- | --- | --- | --- | --- | --- | --- |
|  |  | X | Y | Z |  |  |
| *R Inferior Parietal Lobule* | 353670 | 46 | -46 | 47 | 22.46 | <0.001 |
| R Inferior Parietal Lobule |  | 38 | -55 | 42 |  |  |
| R Superior Occipital Lobule |  | 33 | -64 | 41 |  |  |
| *R Inferior Frontal Gyrus* | 141970 | 33 | 20 | -2 | 20.99 | <0.001 |
| R Anterior Cingulate   Cortex |  | 5 | 19 | 45 |  |  |
| L Supplementary Motor  Area |  | -5 | 17 | 44 |  |  |
| *L Inferior Parietal Lobule* | 288650 | -36 | -47 | 38 | 19.70 | <0.001 |
| L Inferior Parietal Lobule |  | -30 | -57 | 42 |  |  |
| L Inferior Parietal Lobule |  | -39 | -55 | 50 |  |  |
| *R Cerebellum Crus I* | 135570 | 32 | -64 | -34 | 16.45 | <0.001 |
| L Cerebellum Crus I |  | -31 | -65 | -36 |  |  |
| L Cerebellum Crus II |  | -8 | -78 | -33 |  |  |
| *L Putamen* | 3030 | -17 | 0 | 14 | 8.24 | <0.001 |
| L Pallidum |  | -15 | -1 | 3 |  |  |
| L Thalamus |  | -14 | -10 | 0 |  |  |
| *R Inferior Temporal Gyrus* | 885 | 58 | -44 | -15 | 7.47 | <0.001 |
| R Middle Temporal Gyrus |  | 64 | -36 | -10 |  |  |
| R Middle Temporal Gyrus |  | 46 | -30 | -13 |  |  |
| *R Caudate Nucleus* | 4013 | 16 | -2 | 16 | 7.44 | <0.001 |
| R Pallidum |  | 16 | 2 | -1 |  |  |
| R Thalamus |  | 13 | -8 | 1 |  |  |
| *R Cerebellum Vermis* | 7489 | 1 | -47 | -22 | 7.22 | <0.001 |
| L Cerebellum Anterior  Lobe |  | -7 | -51 | -35 |  |  |
| R Cerebellum Anterior   Lobe |  | 5 | -40 | -25 |  |  |
| *L Inferior Occipital Gyrus* | 416 | -35 | -91 | -14 | 5.99 | 0.002 |
| *L Middle Occipital Gyrus* | 558 | -44 | -67 | -16 | 5.73 | 0.001 |

Supplementary Table 3. Clusters that show a quadratic increases in BOLD response within the functionally defined working memory network that is strongest during the 2-back. Cluster p-values are provided and t-statistics for cluster peak coordinates. Local maxima in each cluster more than 8mm apart are noted underneath.

| **Region** | **Size (voxels)** | **MNI** |  |  | **Peak t-value** | **Cluster p-value  (FWE corrected)** |
| --- | --- | --- | --- | --- | --- | --- |
|  |  | X | Y | Z |  |  |
| L Middle Frontal Gyrus | 116052 | -27 | 0 | 52 | 11.86 | <0.001 |
| R Middle Frontal Gyrus |  | 29 | 7 | 52 |  |  |
| L Precentral Gyrus |  | -43 | 0 | 43 |  |  |
| L Inferior Parietal Lobule | 278500 | -36 | -46 | 38 | 11.72 | <0.001 |
| L Superior Parietal Lobule |  | -30 | -64 | 44 |  |  |
| L Inferior Parietal Lobule |  | -38 | -56 | 50 |  |  |
| R Inferior Parietal Lobule | 30202 | 44 | -45 | 48 | 10.97 | <0.001 |
| R Supramarginal Gyrus |  | 41 | -44 | 37 |  |  |
| R Superior Parietal Lobule |  | 32 | -66 | 44 |  |  |
| L Cerebellum Crus I | 10882 | -31 | -65 | -36 | 9.59 | <0.001 |
| R Cerebellum Crus I |  | 30 | -65 | -33 |  |  |
| L Cerebellum Crus II |  | -7 | -79 | -32 |  |  |
| L Putamen | 2702 | -18 | 0 | 15 | 7.64 | <0.001 |
| L Pallidum |  | -18 | 1 | 0 |  |  |
| R Caudate Nucleus | 1076 | 19 | -1 | 16 | 7.23 | <0.001 |
| R Thalamus |  | 15 | -9 | 8 |  |  |
| L Inferior Occipital Gyrus | 556 | -48 | -65 | -15 | 7.22 | 0.001 |
| R Inferior Temporal Gyrus | 94 | 52 | -55 | -16 | 5.50 | 0.017 |
| R Inferior Temporal Gyrus |  | 57 | -47 | -15 |  |  |
| R Cerebellum Vermis | 318 | 0 | -50 | -22 | 5.00 | 0.004 |
| R Globus Pallidus | 201 | 15 | -3 | -3 | 4.89 | 0.008 |
| R Pallidum |  | 19 | 5 | 3 |  |  |

Supplementary Table 4. Clusters that show a linear increase in BOLD response within the functionally defined working memory network where response is stronger during 3-back than 1-back and the 2-back is assumed to lie in between. Cluster p-values are provided and t-statistics for cluster peak coordinates. Local maxima in each cluster more than 8mm apart are noted underneath.

| **Region** | **Size (voxels)** | **MNI** |  |  | **Peak t-value** | **Cluster p-value  (FWE corrected)** |
| --- | --- | --- | --- | --- | --- | --- |
|  | 134500 | X | Y | Z | 20.42 | <0.001 |
| R Middle Frontal Gyrus |  | 27 | 10 | 53 |  |  |
| R Medial Superior Frontal Gyrus |  | 5 | 21 | 43 |  |  |
| L Supplementary Motor Area |  | -6 | 18 | 44 |  |  |
| R Inferior Parietal Lobule | 344740 | 47 | -45 | 41 | 17.30 | <0.001 |
| R Precuneus |  | 12 | -68 | 48 |  |  |
| R Superior Occipital Gyrus |  | 33 | -67 | 40 |  |  |
| L Inferior Parietal Lobule | 267600 | -37 | -50 | 38 | 16.22 | <0.001 |
| L Inferior Parietal Lobule |  | -32 | -60 | 42 |  |  |
| L Precuneus |  | -9 | -68 | 47 |  |  |
| L Cerebellum Crus I | 4821 | -32 | -65 | -35 | 12.65 | <0.001 |
| L Cerebellum Crus II |  | -9 | -79 | -32 |  |  |
| R Cerebellum Crus II |  | 9 | -78 | -31 |  |  |
| R Cerebellum Crus I | 3405 | 33 | -64 | -34 | 11.49 | <0.001 |
| L Putamen | 2845 | -15 | 1 | 15 | 10.55 | <0.001 |
| L Pallidum |  | -15 | 0 | -1 |  |  |
| R Pallidum | 3832 | 15 | 2 | -2 | 9.28 | <0.001 |
| R Caudate Nucleus |  | 14 | 1 | 16 |  |  |
| R Pallidum |  | 15 | 0 | 6 |  |  |
| L Mesencaphalon | 2040 | -4 | -31 | -21 | 7.13 | <0.001 |
| R Mesencaphalon |  | 4 | -30 | -21 |  |  |
| L Substantia Nigra |  | -7 | -15 | -12 |  |  |
| R Inferior Temporal Lobe | 483 | 57 | -42 | -15 | 6.45 | 0.002 |
| R Middle Temporal Lobe |  | 64 | -36 | -10 |  |  |
| R Cerebellum Vermis | 127 | 0 | -48 | -22 | 5.06 | 0.013 |
